# A case of postpartum dermatomyositis with onset at three months: expanding the clinical spectrum

**DOI:** 10.1097/MS9.0000000000002506

**Published:** 2024-08-30

**Authors:** Sandesh Shah, Garima Shrestha, Ujwal Raut, Monasha Vaidya, Ritesh Thapaliya, Radhika Maharjan

**Affiliations:** a Department of Dermatology and Venerology, B and C Medical College and Teaching Hospital, Birtamode; b KIST Medical College and Teaching Hospital, Lalitpur; c B. P. Koirala Institute of Health Sciences, Dharan; d Department of Pathology, B and C Medical College and Teaching Hospital, Birtamode; e Department of Orthopedics, National Trauma Center, Kathmandu, Nepal

**Keywords:** autoimmune, case report, dermatomyositis, postpartum

## Abstract

**Introduction and importance:**

Dermatomyositis (DM) is an autoimmune disorder affecting all age groups, with a higher prevalence in women. Diagnosis typically occurs around age 40. Manifestation can be acute or chronic. Diagnosis relies on clinical, laboratory, electromyography, and histopathological tests. Limited information exists regarding DM’s occurrence during pregnancy and its connection to pregnancy.

**Case presentation:**

A 31-year-old, 3-month postpartum female presented with features of proximal muscle weakness and generalized erythematous itchy rash. The weakness was associated with muscle tenderness. Laboratory evaluation and skin biopsy revealed features consistent with dermatomyositis. Pulmonary pathology and occult tumors were investigated. Symptoms gradually improved on steroids and azathioprine.

**Clinical discussion:**

The relationship between dermatomyositis and pregnancy remains poorly understood, with only a few documented cases highlighting the impact of pregnancy on the development and progression of the disease. From the available literature, it is evident that postpartum dermatomyositis is uncommon yet significant, necessitating careful attention during pregnancy to enhance outcomes for both the mother and the unborn child.

**Conclusion:**

Our experience highlights a rare case of postpartum dermatomyositis, with symptoms emerging 3 months after childbirth, differing from documented cases. More research is needed to understand pregnancy’s role in dermatomyositis development and improve treatment for pregnant women with autoimmune skin diseases.

## Introduction

Highlights

- Dermatomyositis (DM) is an autoimmune disorder affecting all age groups, with a higher prevalence in women.
- A 31-year-old, 3-month postpartum female presented with features of proximal muscle weakness and generalized erythematous itchy rash.
- The relationship between dermatomyositis and pregnancy remains poorly understood.
- Symptoms gradually improved with steroids and azathioprine.

Dermatomyositis (DM), an autoimmune inflammatory disorder, represents an idiopathic inflammatory myopathy. It affects individuals across all age groups, with an approximate prevalence ranging from 5 to 22 cases per 100 000 inhabitants. On average, it is diagnosed around the age of 40, with women getting affected twice as compared to men^1,2^. The manifestation of DM can either be acute, occurring over weeks or chronic, developing over months to years. Clinical, laboratory, electromyography (EMG), and histopathological tests aid in its diagnosis^3^.

Most cases of dermatomyositis during and around pregnancy have been discussed emphasizing the management of high-risk pregnancies, and there is not much information about how dermatomyositis and pregnancy are connected. It is uncertain if dermatomyositis occurs less often during pregnancy and peripartum period, as the rare cases might be due to the uncommon condition among young adult women^4^.

We report a case of a 31-year-old female, previously healthy, who presented with features suggestive of dermatomyositis 3 months postpartum.

Guidelines: SCARE 2023 paper^5^.

This case has been reported in line with the SCARE 2023 criteria.

## Case presentation

### Patient information

#### Demographics and presentation

**Figure 1:**
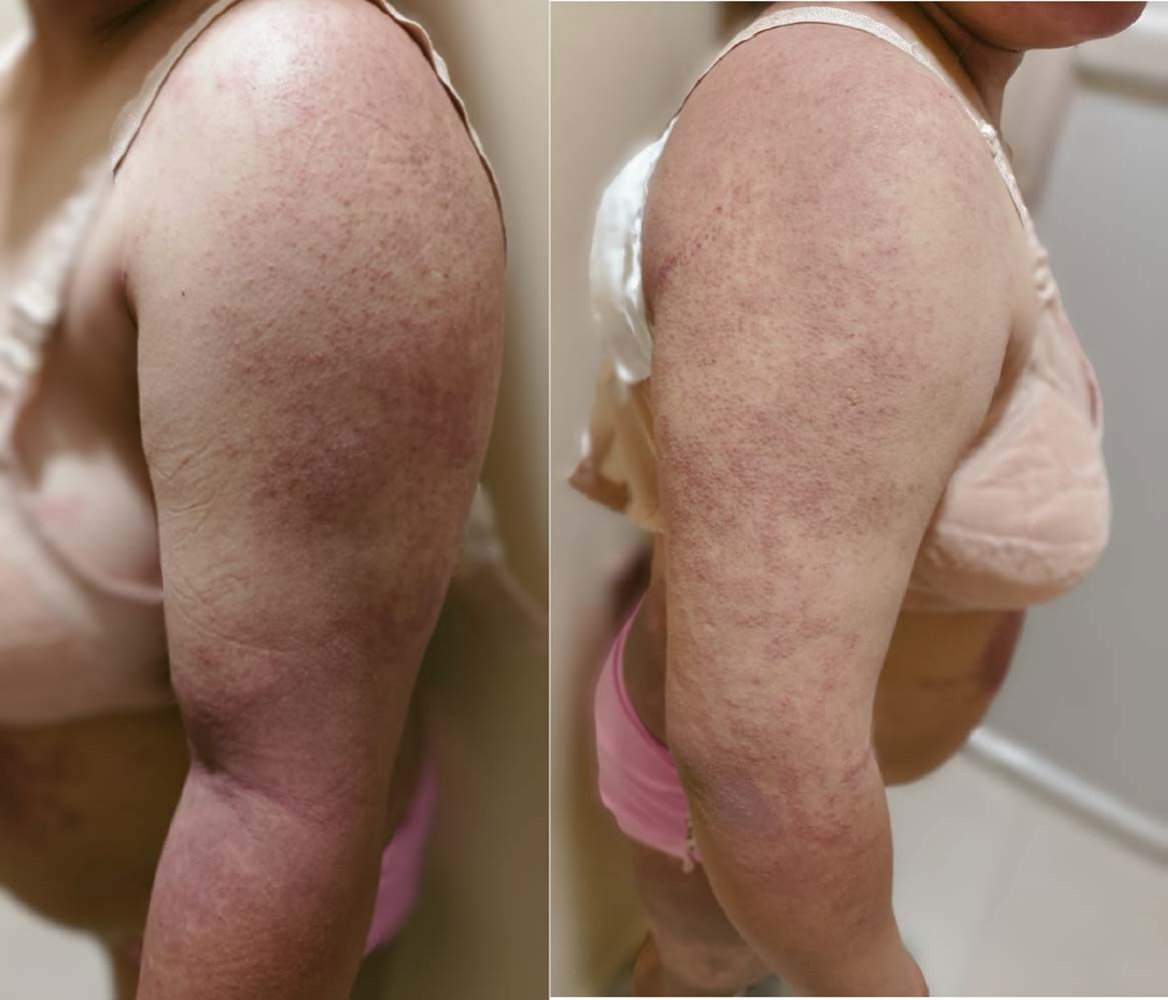
Erythematous to dusky brown plaque over bilateral upper limbs.

**Figure 2:**
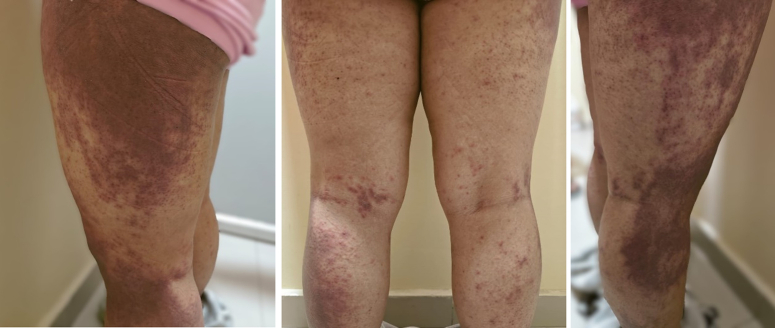
Erythematous to dusky brown plaques over bilateral lower limbs.

**Figure 3:**
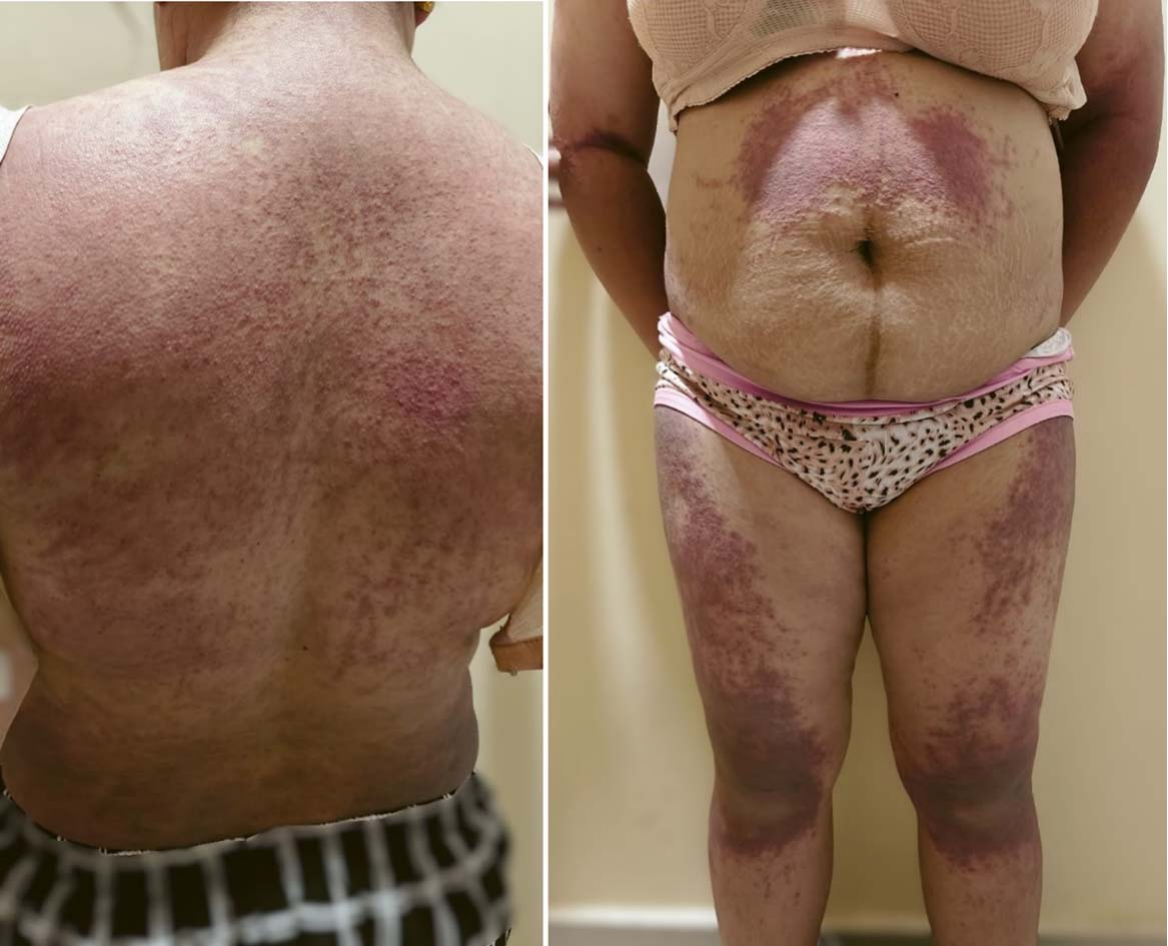
Erythematous to dusky brown plaques over the torso and lower limbs.

**Figure 4:**
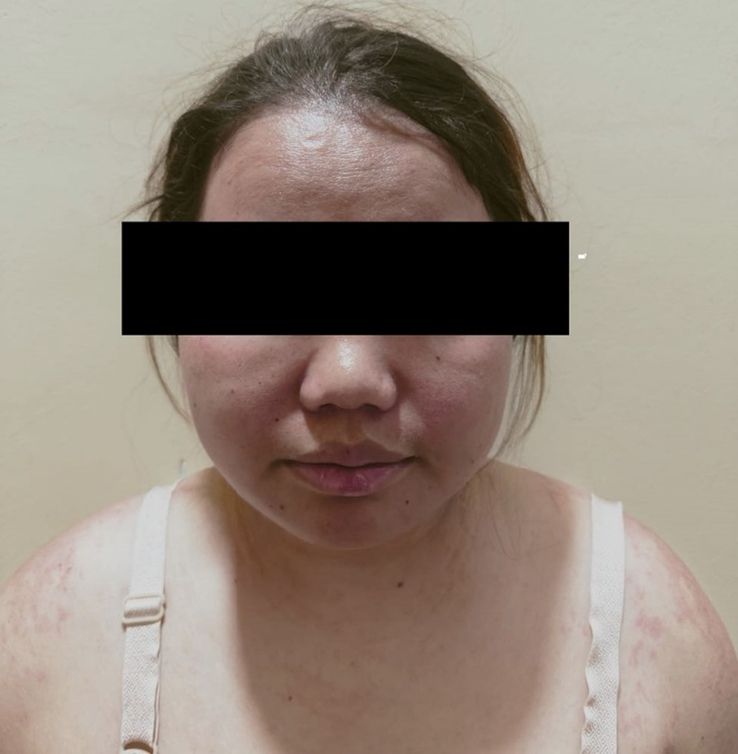
Figure showing facial puffiness along with mild scaling.

A 31-year-old female presented to the clinic 3 months postpartum with complaints of proximal muscle weakness, facial puffiness, and generalized itchy erythematous rash. She reports that her symptoms initially began with a fever of mild grade with no apparent systemic signs or symptoms. Subsequently, she started experiencing weakness in her limbs, which affected her daily activities like standing from a sitting position, walking, combing her hair, and grabbing overhead items with her hand. The weakness was associated with pain in her upper and lower limbs. Serially, she noticed the development of itchy erythematous to dusky brown macules generalized all over the body (Figs 1–3) associated with facial swelling (Fig. 4). The patient reports no relevant family history, past medical or surgical procedures, or current medications or allergies.

### Clinical findings

On presentation, the patient was alert and oriented but appeared fatigued. Apart from a fever, which was documented to be 101°F, her vital signs were stable. She exhibited dusky erythematous violaceous plaques with scaling and mild atrophy on her chest, back, and limbs, with the lower limbs showing a more pronounced rash than the upper. There was no evidence of associated ulcerations or blisters. Facial puffiness with periorbital edema was also noted. Further examination revealed weakness in the proximal group of muscles, with a power rating of 4/5 in both upper and lower limbs, along with normal bulk, tone, and reflexes. Additionally, the proximal muscles of both lower and upper limbs were tender upon palpation. The remainder of the physical examination was unremarkable.

### Diagnostic assessment and interpretation

**Figure 5:**
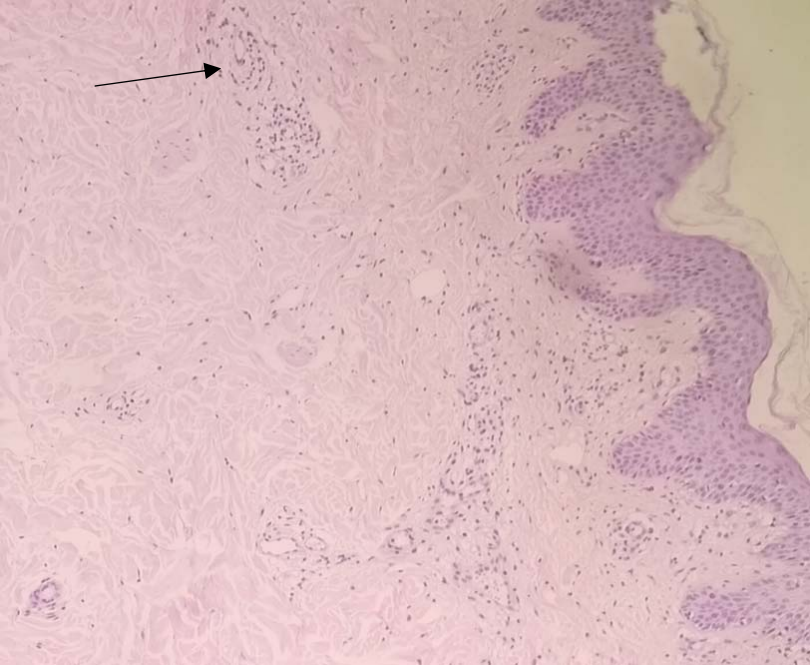
Hematoxylin and eosin stain of skin punch biopsy showing myositis with myofiber necrosis and fragmentation (arrow).

**Figure 6:**
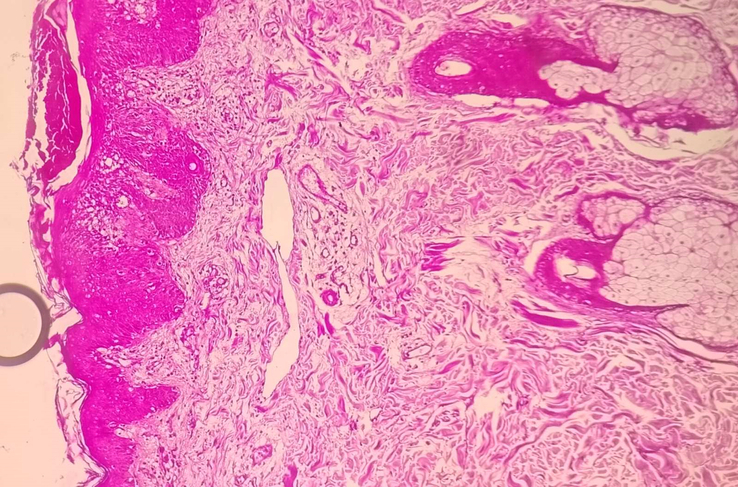
Periodic acid-Schiff (PAS) stain of skin punch biopsy showing abundant dermal mucin.

Laboratory evaluation revealed a positive anti-Jo-1 antibody and elevated levels of creatine kinase (623 U/l), ESR (30 mm), CRP (20 mg/dl), liver enzymes (AST 434 U/l, ALT 234 U/l), and LDH (1455 U/l). The complete blood count and basic metabolic panel were unremarkable. The ANA report indicated greater than 1/1320 with a mottled pattern. A skin biopsy from the plaque over the affected back area revealed findings consistent with dermatomyositis (Figs. 5, 6). In consideration of the clinical, laboratory, and skin biopsy findings, a muscle biopsy was not performed, following a shared decision-making process with the patient. Electromyography (EMG) could not be performed due to unavailability. A final diagnosis of dermatomyositis was made. Chest radiograph and CT of the abdomen/pelvis revealed no pulmonary pathology or occult tumors. Additionally, fecal occult blood test (FOBT) and Pap smear results were negative.

### Intervention and follow-up

She was treated on an outpatient basis as the patient denied inpatient admission and was started on prednisolone and azathioprine, 0.5 mg/kg/day and 50 mg/day, respectively, both in a single dose per day regimen. Additionally, topical tacrolimus, oral antihistamines, and topical coconut oil were prescribed to decrease her pruritus. The patient was advised to follow up after 3 weeks. She reported a 50% improvement in her muscle weakness, evidenced by the ease with which she could perform her daily activities, along with the improvement of her rash. CK levels 3 weeks later were 234 U/l. Currently, the patient is on a tapering dose of oral steroids along with azathioprine 50 mg twice daily. To monitor her condition closely, the patient will be followed up monthly for the next 3 months. These monthly visits will include checking her CK levels, a musculoskeletal examination along with routine blood investigations to assess her muscles and joints, and a general physical examination to ensure her overall health. After this initial 3-month period, recognizing the distance the patient travels, the patient will be followed up every 6 months.

## Discussion

Dermatomyositis is shown to occur more frequently among women, with only 14% of the cases occurring during the reproductive age group, more commonly in the 10–15 years and 45–60 years age groups^6^. There is limited understanding, along with few reported cases, regarding the relationship between dermatomyositis and pregnancy, specifically the potential contribution of pregnancy to the onset and course of the disease^2^.

The literature on postpartum dermatomyositis is limited but highlights the potential complications for both mother and fetus. Tang *et al*.^7^ discuss the challenges in treating autoimmune skin diseases during pregnancy, including dermatomyositis, emphasizing the importance of disease control and minimizing risks to both mother and fetus. Wan *et al*.^8^ mention the scarcity of studies on pregnancy in dermatomyositis patients, mostly limited to case reports or small samples. Missumi *et al*.^9^ emphasize the lack of research on pregnancy outcomes in women with inflammatory myopathies, aiming to evaluate the effects of these conditions on maternal and neonatal outcomes. Tuccinardi *et al*.^10^ report a rare case of dermatomyositis developing after delivery, suggesting that pregnancy could be a trigger for the onset of the disease. They propose two types of pregnancy-related DM, one with disease activity provoked during pregnancy and improving postpartum, and the other with onset in the postpartum period. Kanoh *et al*.^4^ also note the rare occurrence of DM during the reproductive period and its potential adverse effects on fetal outcomes. Lee and Yoo^11^ further support the idea that pregnancy could contribute to the development and course of DM based on a case study and a review of postpartum-onset DM cases. Overall, the literature review suggests that postpartum dermatomyositis is a rare but significant condition that requires careful management during pregnancy to optimize outcomes for both the mother and the fetus.

Further research is needed to better understand the relationship between pregnancy and the development of dermatomyositis, as well as to improve treatment strategies for pregnant women with autoimmune skin diseases^12^.

## Conclusion

Dermatomyositis shows limited understanding regarding its relation to pregnancy. Research highlights potential complications for both mother and fetus, emphasizing the importance of disease control during pregnancy. Studies suggest pregnancy could trigger the onset of dermatomyositis, requiring careful management to optimize outcomes for both mother and fetus. Although postpartum dermatomyositis is frequently missed and misdiagnosed, our case is particularly uncommon. While it typically appears a few months after delivery, this instance highlights the possibility of dermatomyositis emerging just 3 months postpartum. This emphasizes the importance of considering dermatomyositis in postpartum patients with skin rash and muscle weakness. Regarding the limitation, an electromyography (EMG) test would have been ideal for confirmation, but it was not available. However, the patient’s typical symptoms, combined with lab results and a skin biopsy, provided enough evidence for a diagnosis.

## Ethical approval

Not applicable.

## Consent

Written informed consent was obtained from the patient for the publication of this case report and accompanying images. A copy of the written consent is available for review by the Editor-in-Chief of this journal on request.

## Source of funding

This case hasn’t been funded by any person or any institution.

## Author contribution

S.S. and G.S.: conceptualization, methodology, writing – original draft preparation, supervision, and validation; U.R.: conceptualization, writing – original draft preparation, and software; M.V.: visualization, data curation, methodology, and writing – original draft preparation; R.T.: conceptualization, data curation, software, and supervision; R.M.: writing – reviewing and editing, and project administration.

## Conflicts of interest disclosure

The authors declare no conflicts of interest.

## Research registration unique identifying number (UIN)

Not applicable.

## Guarantor

Sandesh Shah, MD, Department of Dermatology and Venerology, B and C Medical College and Teaching Hospital, Birtamode, Nepal.

## Data availability statement

Not applicable.

## Provenance and peer review

Not commissioned, externally peer-reviewed.
